# Immune Responses and Pathogenesis following Experimental SARS-CoV-2 Infection in Domestic Cats

**DOI:** 10.3390/v15051052

**Published:** 2023-04-25

**Authors:** Sandra Vreman, Elisabeth M. D. L. van der Heijden, Lars Ravesloot, Irene S. Ludwig, Judith M. A. van den Brand, Frank Harders, Andries A. Kampfraath, Herman F. Egberink, Jose L. Gonzales, Nadia Oreshkova, Femke Broere, Wim H. M. van der Poel, Nora M. Gerhards

**Affiliations:** 1 Wageningen Bioveterinary Research, Wageningen University & Research, 8221 RA Lelystad, The Netherlands; 2 Division of Infectious Diseases and Immunology, Department of Biomolecular Health Sciences, Faculty of Veterinary Medicine, Utrecht University, 3584 CL Utrecht, The Netherlands; 3 Division of Pathology, Faculty of Veterinary Medicine, Utrecht University, 3584 CL Utrecht, The Netherlands

**Keywords:** cats, SARS-CoV-2, immunology, pathology, viral shedding, COVID-19, intranasal inoculation, CD4 and CD8

## Abstract

Several reports demonstrated the susceptibility of domestic cats to SARS-CoV-2 infection. Here, we describe a thorough investigation of the immune responses in cats after experimental SARS-CoV-2 inoculation, along with the characterization of infection kinetics and pathological lesions. Specific pathogen-free domestic cats (*n* = 12) were intranasally inoculated with SARS-CoV-2 and subsequently sacrificed on DPI (days post-inoculation) 2, 4, 7 and 14. None of the infected cats developed clinical signs. Only mild histopathologic lung changes associated with virus antigen expression were observed mainly on DPI 4 and 7. Viral RNA was present until DPI 7, predominantly in nasal and throat swabs. The infectious virus could be isolated from the nose, trachea and lungs until DPI 7. In the swab samples, no biologically relevant SARS-CoV-2 mutations were observed over time. From DPI 7 onwards, all cats developed a humoral immune response. The cellular immune responses were limited to DPI 7. Cats showed an increase in CD8+ cells, and the subsequent RNA sequence analysis of CD4+ and CD8+ subsets revealed a prominent upregulation of antiviral and inflammatory genes on DPI 2. In conclusion, infected domestic cats developed a strong antiviral response and cleared the virus within the first week after infection without overt clinical signs and relevant virus mutations.

## 1. Introduction

The global SARS-CoV-2 (Severe Acute Respiratory Syndrome Coronavirus 2) pandemic is turning endemic, and at this time, many countries have reduced or revoked their COVID-19 (Coronavirus Disease 19)-associated restrictions [1]. However, awareness for SARS-CoV-2′s capacity to become a health emergency is still warranted and attention is needed for the formation of potential animal reservoirs with associated virus adaptations. The susceptibility of animals has been demonstrated in experimental studies of, e.g., ferrets, cats, hamsters and non-human primates [2,3]. Moreover, a variety of animal species can be naturally infected after contact with infected humans, as reported for minks, dogs, domestic and big cats and hamsters, amongst others [4,5].

Domestic cats are of special interest, because they are often in close contact with their owners, other cats of the same household and cats in the neighborhood. Research has demonstrated that there is viral transmission between infected cats [6], and it has been suggested that cats could play a role in the maintenance and transmission of SARS-CoV-2 [7]. In experimental studies, SARS-CoV-2-infected cats showed mostly mild clinical signs and mild pathological changes in the lungs [8]. Next to clinical signs and pathology, the evaluation of the protective immune response after infection is of importance. Humoral antibody-related immune responses are generally evaluated in studies [9], but to our knowledge, there is only limited information about T-cell-induced immune responses in felines shortly after SARS-CoV-2-induced infection. Antiviral CD4+ T cells could be important for the induction of antibody responses and IFN-gamma production, while cytotoxic CD8+ cells are needed to reduce the viral load in infected individuals [10,11]. Both cell subsets are important parts of an antiviral immune response.

To assess the characteristics of cats’ immune response and associated pathogenesis, twelve domestic cats were intranasally infected with SARS-CoV-2, and their humoral and T-cell-mediated antiviral responses were evaluated over time in relation to the viral loads, viral mutation and pathological changes. We showed that the infected domestic cats developed a strong antiviral immune response with rapid clearance of the virus within the first week after infection without overt clinical signs and relevant virus mutations.

## 2. Materials and Methods

### 2.1. Ethical Statement, Animal Housing and Experimental Design

The study (experiment number 2020.D-0007.010) was performed under legislation of the Dutch Central Authority for Scientific Procedures on Animals (CCD license no. AVD4010020209446) and approved by the Animal Welfare Body of Wageningen University and Research prior to the start of the in-life phase. Animal and laboratory work were performed in human biosafety level 3 laboratories (hBSL3) at Wageningen Bioveterinary Research, Lelystad (WBVR), The Netherlands, and additional laboratory work was performed after viral inactivation at the Faculty of Veterinary Medicine, Utrecht University, Utrecht, The Netherlands.

Fourteen cats (seven male and seven female domestic shorthair cats, body weight range 2.0–3.5 kg, 4 months of age upon arrival from Marshall Bioresources (Waverly, NY, USA) and vaccinated against rabies lyssavirus and feline herpesvirus, feline calicivirus and feline panleukopenia virus) were included in this study. The cat colony was free of feline coronavirus, calicivirus, herpesvirus, panleukopenia virus, feline leukemia virus and feline immunodeficiency virus, as stated by the breeder’s health certificate (Supplemental Figure S5). Prior to SARS-CoV-2 inoculation, the absence of antibodies against SARS-CoV-2 was confirmed by microneutralization and the ELISA assay (described under Section 2.6), and the absence of SARS-CoV-2 nucleic acid was confirmed by RT-PCR with oral, rectal, nasal and throat swabs (see Section 2.4). Cats arrived on DPI (day post-inoculation) −18 to acclimatize to the new environment for 11 days. The acclimatization period also served as the socialization period, in which animal caretakers handled the cats daily to acquaint them with the protective overalls, hBSL3-level respirators, litter boxes and all daily handlings that were scheduled during the course of the experiment. Cats received a subcutaneous chip for identification linked to an animal ID, which was also written on a collar. No blinding of personnel took place. On DPI −7, a temperature transponder (Anipil, Animals Monitoring, Hérouville Saint-Clair, France) was implanted in the abdominal cavity of all cats under general anesthesia after intramuscular (IM) injection of medetomidine (0.04 mg/kg) and ketamine (4 mg/kg), which was antagonized by atipamezole IM (0.1 mg/kg). All cats received meloxicam orally (0.5 mg/kg) on the day of surgery and the following day. All medication was obtained from AST Farma, Oudewater, The Netherlands.

The cats were randomized using the randomizer function in Microsoft Excel based on sex and body weight into two groups: one experimentalgroup with twelve SARS-CoV-2-infected cats (*n* = 12; seven males and five females) and a control group with two uninfected control cats (*n* = 2 females). The two control cats were housed together in one separate animal unit; while the remaining twelve cats were housed in two adjacent pens, separated by sex, in a second animal unit. The control group was housed under veterinary BSL2 level throughout the experiment, whereas the experimental group was housed under human BSL3 conditions post-inoculation. After inoculation on DPI 0 with SARS-CoV-2, each three animals from the infected group were subsequently sacrificed on DPI 2, 4, 7 and 14. The uninfected control animals were sacrificed on DPI 14 (Overview in Supplemental Table S1).

Cats were housed with 12 h of light and 12 h of darkness per day and were provided with water and commercial cat pellets ad libitum. On the evenings before the days the cats underwent anesthesia, the feed was removed from the pens. Hammocks, baskets, elevated resting spots, pillows, towels, toys and scratching posts were available as enrichment.

### 2.2. Virus, Animal Inoculation and Sampling

SARS-CoV-2/human/NL/Lelystad/2020 (GenBank accession number MZ14458) was produced on Vero E6 cells and used undiluted for the challenge inoculation, as described previously [12].

On DPI 0, twelve cats were inoculated intranasally with 10^4.5^ TCID_50_ SARS-CoV-2 under general anesthesia in a volume of 0.5 mL (0.25 mL per nostril synchronous with the cat’s breathing rhythm). The uninfected control animals were inoculated intranasally with 0.5mL PBS under general anesthesia. After inoculation, the cats were observed daily for their general health and respiratory signs post-challenge. Body temperature was measured every hour with the intra-abdominal Anipil. Body weight was measured on DPI −17, −14, −12, −10, −7, −5, −3, 0, 2, 4, 7, 9, 11 and 14. All cats were anesthetized as described earlier on DPI 0, 2, 4, 7, 9, 11 and 14 for sampling. Cats were euthanized on different days post-challenge inoculation to collect post-mortem samples. Blood samples (2 mL serum and 1mL heparinized blood), nasal swabs and throat swabs were obtained at DPI 0, 2, 4, 7, 9, 11 and 14 under general anesthesia. Rectal/fecal samples were taken daily from DPI 0 until the end of the study, except for DPI 7.

Electrostatic dust cloths were used to sample the environment daily from DPI 0 to DPI 14. There were four different sample locations: floor (random area of 10 × 10 cm), wall (random area of 1 × 1 m), feeding tray (one feeding tray was wiped before feed was added) and toy (a random toy was wiped). Samples were taken alternating from the pen with females or with males (DPI 0, 2, 4, 6, 8, 10, 12 and 14 = females; DPI 1, 3, 5, 7, 9, 11 and 13 = males).

The air of the animal unit in which the inoculated group was housed was sampled daily post-challenge inoculation. A liquid cyclonic collector (Midwest Micro Tek) [13] was positioned in the animal unit outside of the animals’ pens at approximately 1.5 m in height. The sampler’s flow rate was set to 300 L/min, and 10 mL of MEM cell culture medium was used as the collection media. After 30 min of sampling, approximately 4 mL collection medium could be recovered.

Exhalation air samples were collected using a MD8 Airport Portable Air Sampler (Sartorius, Goettingen, Germany). Sedated cats were placed closely in front of a gelatin filter (3 μm pore size) attached to a MD8 sampler for 3 min at a 5.0 m^3^/h flow rate. Exhale samples were collected from inoculated cats on the days of anesthesia or necropsy (DPI 0 (*n* = 12), 2 (*n* = 12), 4 (*n* = 9), 7 (*n* = 6), 9 (*n* = 3), 11 (*n* = 3) and 14 (*n* = 3)).

### 2.3. Gross and Histological Examination and Immunohistochemistry

According to the experimental design, on DPI 2, 4, 7 and 14 (Supplemental Table S1), cats were euthanized to perform necropsy with a full macroscopic examination of all major organs and to collect blood and tissue samples. Euthanasia was performed by the intramuscular injection of earlier described anesthetics, followed by blood sampling through the aorta and exsanguination through the brachiocephalic vein. The lungs were weighed after exsanguination to be expressed as a percentage of the body weight. The complete left lung was instilled with 4% neutral buffered formalin for histology. From every left lung lobe (cranial, medial and caudal), 2 sections, a transverse and a longitudinal section, were evaluated. The following other organs were sampled in formalin for histopathology and immunohistochemistry (IHC): nasal conchae; trachea; intestinal tract (duodenum, pancreas, ileum, colon and mesenterial lymph node); spleen; liver; heart; kidney; urinary bladder; cheek; larynx, eye and brain (cerebrum, cerebellum and olfactory bulb). Formalin-fixed samples were embedded in paraffin, sectioned at 5 μm and stained with hematoxylin and eosin (H&E) for histological examination, according to the general pathology principles. IHC of histological specimens was performed with an antibody directed against the SARS-CoV nucleoprotein, as previously described [12].

### 2.4. Virology: Sample Processing, E-Gene PCR, Subgenomic PCR and Virus Isolation

Respiratory organs (nasal conchae, trachea and lungs); salivary gland and intestinal tract (duodenum, ileum and colon) were stored at ≤ −80 °C until further processing. Each organ sample was weighed and subsequently homogenized in 6mL MEM with 1% antibiotic/antimycotic solution (both from Gibco; Thermo Fischer Scientific; Waltham, MA, USA) in an Ultra Turrax Tube Drive (IKA; Staufen, Germany) for 30 s (lungs) or 50 s (conchae) at 6000 rpm. The obtained homogenates were centrifuged at 3400× *g* at 4 °C for 15 min. Two aliquots of the cleared suspension were frozen at ≤−80 °C for virus isolation, and one aliquot was mixed 1:3 with Trizol-LS (Sigma; St. Louis, MO, USA) and stored at ≤−20 °C until RNA isolation. Virus isolation of E-gene-PCR-positive samples from the respiratory tract was performed on Vero E6 cells as described previously [12]. Environmental tissues (floor, wall, feeding tray and toy) were collected in individual seal bags and frozen directly at −80 °C. To recover viral RNA, 10mL of tissue culture medium (MEM) was added to the tissues and squidged vigorously, before taking a sample of 85 µL in 255 µL Trizol-LS for RNA isolation.

For cyclone air samples, 85 μL of the recovered sample was added to 255 μL Trizol-LS and used for RNA extraction. RNA was eluted in 50 μL and analyzed by E-gene PCR. Exhalation air filters were thawed at room temperature and dissolved in 10 mL MEM, which was prewarmed at 37 °C. After the gelatin was dissolved, 200 μL sample was added to 600 μL Trizol-LS for RNA extraction. After extraction, the eluate was concentrated to a volume of 15 μL using a RNA Clean and Concentrator kit (Zymo Research, Irvine, CA, USA) before analysis by E-gene PCR. For both types of air samples, RNA copy numbers were calculated as following: Copies/L = (copies/mL ∗ volume of sample in mL)/(sampler flow rate (L/min) ∗ sampling time (min)).

Swabs were submerged in 2 mL MEM directly after sampling, kept on melting ice before vigorously vortexing for 30 s on a vortex (Labdancer, VWR International B.V., Amsterdam, The Netherlands). After centrifugation for 5 min at 1500× *g* and 4 °C, a sample aliquot of 200 µL was mixed with 200 µL lysis buffer (Molgen, Veenendaal, The Netherlands), and stored at ≤−20 °C until RNA isolation. 

RNA of samples stored in Trizol-LS was extracted using Direct-zol™ RNA MiniPrep kit (Zymo Research, RefNo R1013; Irvine, CA, USA) according to manufacturer’s instructions, without DNase treatment. RNA of samples stored in Molgen lysis buffer was isolated by an automated robot system (PurePrep 96) using the Molgen RNA isolation kit (OE00290096). Isolated RNA was kept at −80 °C and analyzed for the presence of SARS-CoV-2 E-gene and subgenomic RNA according to [14,15]. PCR results were expressed as Log_10_ viral RNA copies, based on standard curves, as described previously [12].

### 2.5. Viral Sequencing and Variant Analysis

Organ, swab and environmental dust cloth samples which yielded a Ct value ≤35 by E-gene PCR were selected for viral sequencing. RNA was extracted from another aliquot that was stored at ≤−80 °C using the Direct-Zol RNA Miniprep kit (Zymo Research, Irvine, CA, USA) according to manufacturer’s protocol including DNase treatment after a benzonase treatment, followed by the RNA clean and concentrate kit (Zymo Research, Irvine, CA, USA).

Filtering, mapping and variant calling of the 78 sequenced samples and inoculum was done with the Nextflow core pipeline viralrecon (v. 2.5; https://nf-co.re/viralrecon/2.5, accessed on 7 November 2022) from a docker container, following the protocol for samples Illumina sequenced with ARTIC 3 amplicon primers and the SARS-CoV-2 Wuhan-Hu-1 strain as reference genome (GenBank accession MN908947.3). A sample sheet was created as described on the nf-core/viralrecon website (https://nf-co.re/viralrecon/usage, accessed on 7 November 2022). Since assemblies were already available, this part of the pipeline was skipped, and thresholds for the iVar variant caller (v.1.3.1) were adjusted to 0.03 for minor variants and 0.97 for calling consensus by adding a custom config file to the pipeline, as these cut-offs were used in for a comparable SARS-CoV-2 study [16] and are advised based on a study searching for the best settings of iVar on amplicon based sequenced samples [17]. Called variants (n = 2652) were filtered for failing Fisher’s exact test in variant frequency compared to mean error rate with a *p*-value > 0.05 and in cats and environmental samples were filtered for variants found within the inoculum, because the latter were already present in the inoculum and did not arise as a response to the interaction with the cat. The frequencies of the resulting 1766 variants were plotted over their position on genome per cat in R (v.4.2.0) [18] using the ggplot2 package (v.3.3.5) [19] (Supplemental Figure S3).

### 2.6. Detection of Anti-S1 and Neutralizing Antibodies

Collected serum blood samples (DPI 0, 2, 4, 7, 11 and 14) were separated by centrifugation for 10 min at 1250× *g* at RT after clotting for at least 1h at RT. After heat inactivation for 2h at 56 °C, the resultant sera were stored at ≤−20 °C. Anti-S1 ELISA of sera was performed as described previously [20]. 

Wildtype virus neutralization tests (VNT) and immuno-Peroxidase Monolayer Assay (IPMA) were performed in technical duplicates by 3-fold serial dilutions on 96-well plates, as described previously [12]. The serum was initially diluted 1:10, and 50 µL of each sample were added to 50 µL SARS-CoV-2 (~100 TCID_50_) in MEM. After an incubation step of 1.5 h, 15,000 Vero-E6 cells were added to each well and incubated for 4 days after which the plates were fixed with 4% formaldehyde and permeabilized with ice-cold 100% methanol. The plates were furthermore permeabilized with 1% Triton X-100 and blocked with 5% normal horse serum in PBS. A rabbit antiserum (rabbit-anti-SARS-CoV-2-S1-2ST (619 F), David’s Biotechnologie GmbH, Regensburg, Germany), followed by goat-anti-rabbit-HRP (Dako; Agilent; Santa Clara, CA, USA) and AEC (3-Amino-9-ethylcarbazole) substrate solution, was applied to detect the viral spike protein. A clear red-brown color was subsequently evaluated under a standard light microscope. The titer of each duplicate was calculated as the average of the reciprocal value of the last dilution that showed at least 50% neutralization, after log transformation. The titers are expressed as virus microneutralization titer 50 (MN_50_).

### 2.7. Cellular Immune Responses: T-Cell Subsets and Interferon-γ-ELISpot

Five hundred microliters of heparinized whole blood (DPI 0, 4, 7, 11 and 14) was added to forty-five hundred microliters of ACK lysis buffer and incubated for 5 min at room temperature. After a centrifugation step (7 min, 400× *g*), the pellet was resuspended in 500 µL FACS buffer (PBS, Thermo Fischer Scientific, Waltham, MA, USA supplemented with 10% FCS, Thermo Fischer Scientific, Waltham, MA, USA). 100 µL cell suspension was added per well to a 96-wells plate before centrifugation for 4 min at 400× *g*. Cells were incubated with anti-CD4-PE (clone 3-4F4,1:150 dilution, Invitrogen, Waltham, MA, USA) and anti-CD8-BIO (clone fCD8, 1:100 dilution, Invitrogen, USA) antibodies for 30 min on ice. After washing twice in FACS buffer, cells were incubated with streptavidin V500 antibody (1:200 dilution, Bd Biosciences, Franklin lakes, NY, USA) for 30 min on ice. Cells were washed twice in FACS buffer and subsequently fixed with 4% paraformaldehyde (PFA fromWBVR Mediabereiding, Lelystad, The Netherlands) for 30 min on ice. After centrifugation, cells were resuspended in 1% PFA and stored at 4 °C until analysis on a flow cytometer (CytoFLEX, Beckman Coulter Inc., Brea, CA, USA). Data were analyzed using FlowJo Software (FlowJo LCC, Ashland, OR, USA).

During necropsy, 15 mL of heparinized blood was collected from infected animals and, on DPI 14, from 2 uninfected animals to isolate peripheral blood mononuclear cells (PBMCs). Heparinized blood, diluted 1:1 with MEM, was added to leucosep tubes (Greiner Bio-One B.V., Alphen aan den Rijn, The Netherlands) containing ficoll (GE Healthcare Bio Sciences B.V., Danderyd, Sweden) before centrifugation at 1000× *g* for 10 min. The obtained interface was harvested and diluted 1:1 with MEM. After centrifugation for 7 min at 400× *g*, cells were incubated with 4.5mL ACK lysis buffer for 5 min at room temperature and spun down again (7 min, 400× *g*) to obtain a cell pellet which was resuspended in 10 mL MEM. Some (2 × 10^5^) cells were seeded onto an ELISpot plate containing 0.1 mL MEM with or without stimulus in triplicate according to manufacturer’s instructions (Cat IFN-γ ELISpot Plus Kit, MABTech, Nacka Strand, Sweden). The following stimuli were used: Spike protein 30 µg/mL (SARS-CoV-2 (2019-nCoV) Spike Protein (S1+S2 ECD, His tag), Sino Biological, Eschborn, Germany), nucleoprotein 10µg/mL (SARS-CoV-2 (2019-nCoV) NucleocapsidProtein (His tag), Sino Biological, Eschborn, Germany), SARS-CoV-2 Spike protein peptide pool 6µg/mL (Pepscan Presto, Lelystad, The Netherlands) and concanavalin A 20 µg/mL (Sigma, Saint Louis, USA) as a positive control. After incubation at 37 °C and 5% CO_2_ for 48 h, spots were developed and analyzed on a VSpot Spectrum ELISpot reader (AID GmbH, Strassberg, Germany).

Isolated PBMCs from DPI 7 and 14 were also incubated on a 96-well plate (2 × 10^5^ cells/well) with medium only (100 µL) or in the presence of Spike protein (10 µg/mL) in duplicate. After 48 h at 37 °C + 5% CO_2_, cells were harvested and stained with anti-CD4-PE (clone 3-4F4, 1:150 dilution, ThermoFisher Scientific, Waltham, MA, USA) and anti-CD8-BIO (clone fCD8, 1:100 dilution, ThermoFisher Scientific, Waltham, MA, USA) antibodies for 30 min at 4 °C. After washing twice in FACS buffer, cells were incubated with streptavidin V500 (1:200 dilution, BD Biosciences, Franklin lakes, NY, USA) for 30 min at room temperature. Cells were washed twice in FACS buffer. After centrifugation, the cells were resuspended in 1% PFA and stored at 4 °C until analysis on a flow cytometer (CytoFLEX, Beckman Coulter Inc., Brea, CA, USA). Data was analyzed using FlowJo Software (FlowJo LCC, Ashland, OR, USA).

### 2.8. RNA Sequencing of CD8+ and CD4+ Subsets

Isolated PBMCs from all cats were further processed to generate a specific CD4+ and CD8+ subset. In short, PBMCs were centrifuged for 7 min at 400× *g* after which the pellet was incubated with anti-CD4-PE (clone 3-4F4, 1:75) and anti-CD8-BIO (clone fCD8, 1:50) for 30 min on ice. After washing with FACS buffer cells were incubated with streptavidin microbeads (Miltenbiotec, Bergisch Gladbach, Germany) 20 min on ice. After another wash with FACS buffer, CD4+ cells were isolated by passing the cells over a MACS LS-column (Miltenbiotec, Bergisch Gladbach, Germany). Both the flow through and the eluted CD4+ fraction were centrifuged for 7′ at 400× *g*. The pellet from the flow through was incubated with Avidin microbeads (Miltenbiotec, Bergisch Gladbach, Germany) for 20 min on ice after which it was washed with FACS buffer and added to a MACS LS-column. The samples were washed twice with FACS buffer after which the CD8+ cells were eluted and centrifuged for 7 min at 400× *g*. Both the CD4+ and CD8+ cell pellets were resuspended in Trizol (Thermo Fischer Scientific, Waltham, MA, USA) and stored at −80 °C. Total RNA (500 µL) extracted from these subsets was used for library preparation and Illumina sequencing by GenomeScan (Leiden, The Netherlands) using the Takara SMARTer Stranded Total RNA-Seq Kit v3-Pico Input Mammalian Library Prep Kit and NovaSeq6000 (paired-end; 150bp). Festuca [21] was used with default settings to assess read quality. TrimGalore [22] (-q 30 --fastqc) was used to remove adapter sequences and reads of insufficient quality. Next, reads were aligned to the Felis catus 9.0 (felCat9.0; Ensembl v.107 [23]) reference transcriptome using Kallisto (-b 100) [24]. Using R (v.4.2.0 [18]) in R studio (v.7.1 [25]), transcript-level abundances from Kallisto were imported and summarized into counts on gene-level with tximport [26]. For differential expression analysis of genes at the various DPI compared to uninfected animals, DESeq2 [27] was used. Genes were considered differentially expressed when the B-H-adjusted *p*-value was ≤0.05 and the log 2-fold change (L2FC) was |≥2|. Heatmaps and volcano plots were produced using the ComplexHeatmap [28], ggplot2 [19], and ggrepel [29] R packages. The Database for Annotation, Visualization and Integrated Discovery (DAVID) Bioinformatics Resource [30,31] was used for functional annotation analysis of gene ontology (GO) terms significantly differentially expressed genes (DEGs) for selected timepoint comparisons. Only GO terms with a B-H-adjusted *p*-value of ≤ 0.05 were considered significant. The data presented in this study are openly available in the Sequence Read Archive (SRA) of the National Center for Biotechnology Information (NCBI) under BioProject ID PRJNA942620.

## 3. Results

### 3.1. Clinical Signs, Pathological Changes and Immunohistochemistry

Twelve cats were intranasally inoculated with SARS-CoV-2 (SARS-CoV-2/human/NL/Lelystad/2020) and two PBS-inoculated animals were used as uninfected controls in this study. Both male and female cats were investigated in this study, and no clear differences were observed between both sexes. Infected animals were sacrificed at different time-points after infection (DPI 2, 4, 7 and 14, *n* = 3 per time-point) to investigate the pathogenesis and immune responses until DPI 14 and the control animals (*n* = 2) were sacrificed on DPI 14 (Supplemental Figure S1). None of the cats developed any SARS-CoV-2 related respiratory clinical signs after infection. There was a mild rise in body temperature in SARS-CoV-2 inoculated cats between DPI 0 to DPI 4 with the highest average body temperature on DPI 2 (38.9 ± 0.2 °C). The number of white blood cells (WBC) and body weights remained constant during the experiment in the inoculated group as well as in the control group. During necropsy, none of the cats showed SARS-CoV-2 related macroscopic changes neither where there any other relevant changes, that could have influenced the study outcome. The lung weight to body weight ratio was comparable between infected and uninfected control animals (Supplemental Table S2).

**Figure 1:**
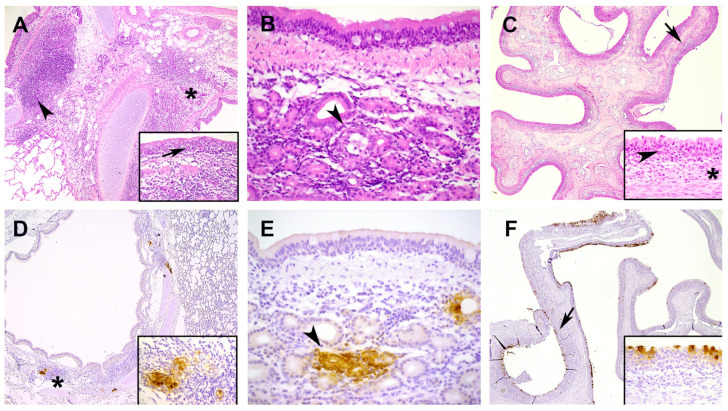
Histopathological features and viral nucleoprotein expression. upper row (**A**–**C**) hematoxylin and eosin stain (HE); bottom row (D-F) SARS-CoV-2 N-protein expression with immunohistochemistry (IHC); (**A**) lung, bronchus (days post-inoculation (DPI) 7), multifocal inflammatory cells in and around submucosal glands (asterisk) with degeneration of lining bronchial-epithelial cells (arrow, insert) and presence bronchiolar alveolar lymphoid tissue (BALT) (arrowhead); objective 10×; (**B**) trachea (DPI 4), presence of mononuclear inflammatory cells and lesser neutrophils in and around submucosal gland with epithelial degeneration (arrowhead), objective 40×; (**C**) nose DPI 7), submucosa is infiltrated with inflammatory cells (neutrophils and lesser macrophages and lymphocytes, arrow) objective 5×, insert: respiratory epithelial lining is infiltrated with inflammatory cells (arrowhead) and inflammatory cells in submucosa (asterisk); (**D**) lung, bronchus (DPI 7) viral antigen expression in epithelial cells of submucosal glands (asterisk), objective 10×, insert location asterisk; (**E**) trachea (DPI 4); viral antigen expression in epithelial cells of submucosal glands (arrowhead), objective 40×; (**F**) nasal conchae (DPI 7) viral antigen expression in nasal respiratory epithelial cells (arrow), objective 5×, insert location arrow; all inserts were made with objective 40×.

Histopathological examination revealed a mild bronchoadenitis in the lungs of the inoculated cats with prominent bronchial alveolar lymphoid tissue (BALT). The bronchoadenitis was characterized by infiltrates of a moderate number of mononuclear cells (mainly macrophages, but also lymphocytes and plasma cells) and fewer neutrophils with loss/degeneration of epithelial cells of the submucosal glands and minimal degeneration of lining bronchial-epithelial cells (Figure 1A). These changes were most prominent on DPI 4 and 7 and were observed in 5 out of the 6 cats sacrificed at these time-points (Supplemental Table S3). In addition, mild to moderate mononuclear to neutrophilic submucosal infiltrates were present in trachea and nasal conchae with also degeneration of epithelial cell of the submucosal glands. These histopathological changes in trachea and nasal conchae were also most prominent on DPI 4 and 7 (Figure 1B,C). On DPI 14 there were no relevant histopathological lesions in the nasal conchae, trachea and lungs of the infected animals Supplemental Table S3). The control animals showed no relevant histopathological changes in the respiratory tract and lungs. None of the cats showed changes in other investigated organs.

Viral antigen expression, measured with immunohistochemistry (IHC), was found in lung (DPI 4 and D7), conchae (DPI 2, 4 and 7) and trachea (DPI 2 and 4) (Figure 1D–F and Supplemental Table S3) in nearly all animals sacrificed at these time-points. The positive anti-NP staining was found in the submucosal glands in the trachea and in the submucosal glands around the large bronchi of the lung. In the nasal conchae, viral antigen expression was found in epithelial cells, but not in the submucosal glands as observed for trachea and lung. For all animals, the intestine (duodenum, ileum and colon); mesenterial lymph node; pancreas; larynx and cheek were evaluated: We found only minimal viral antigen expression in the epithelial and mononuclear cells within the tonsil (larynx) in one animal on DPI 4 and two animals on DPI 7. Spleen; liver; heart; kidney; urinary bladder; eye and brain (cerebrum, cerebellum and olfactory bulb) were evaluated for inoculated animals euthanized on DPI 4 and control animals on DPI 14, and no viral antigen expression was found in these tissues.

### 3.2. Viral Shedding, Environment and Viral RNA Load in Organs

**Figure 2:**
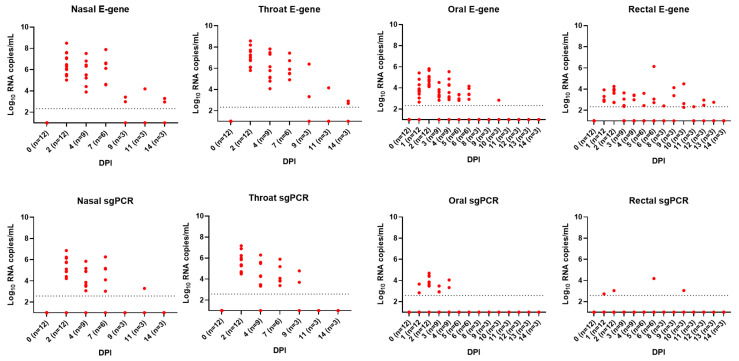
E-gene and subgenomic PCR results on swabs. For E-gene PCR (upper panel) and subgenomic (sg) PCR (lower panel), the log10 RNA copies/mL are shown. All samples for uninfected control animals were negative and these were not included; dotted line indicates PCR detection limit.

Nasal and throat swabs (DPI 0, 2, 4, 7, 9, 11 and 14 collected from sedated cats) and oral and rectal swabs (DPI 0-6 and 8-14 sampled without sedation) were obtained to measure virus shedding by detection of viral RNA (total E gene PCR, which detects genomic and subgenomic (sg) RNA and sg PCR, which detects only sg RNA and is indicative for viral replication). Viral RNA was detected in all infected animals (Figure 2). Already on DPI 1 viral RNA was detected in oral and rectal samples and was still present at the end of the experiment (DPI 14), except for oral swabs (until DPI 10). From DPI 2 until DPI 7, all throat and nasal swabs from infected animals contained viral E-gene and subgenomic RNA, while, on DPI 9 (throat swab) and DPI 11 (nasal swab), subgenomic RNA was only found in a few animals. In oral and rectal swabs, there was variation in the presence of viral RNA between animals, and only a limited number of rectal swabs were sgPCR-positive (Supplemental Table S2).

After necropsy, respiratory organs (nasal conchae, trachea and lungs); salivary gland and intestines (duodenum, ileum and colon) were analyzed for viral RNA by total E gene PCR (Supplemental Figure S2) and sgPCR (Supplemental Table S2) (*n* = 3 per time-point). Consistent with nasal swabs, viral RNA was detected in nasal conchae from DPI 2 until the end of the study and until DPI 7 in the lungs and trachea. No viral RNA was found in salivary glands. In the intestinal samples, the most consistent number of viral RNA copies was found in the ileum and persisted until the end of the study. Within the colon and the duodenum, the presence of viral RNA was more variable. Nearly all E gene-positive nasal conchae and trachea samples also tested positive by sgPCR (Supplemental Table S2). In contrast, only two samples of intestinal tract also tested positive by sgPCR. Virus isolation was performed on respiratory tissues (Supplemental Table S2). The infectious virus was found in trachea and lung on DPI 2 and 4 and in the nasal conchae on DPI 2, 4 and 7 but not in all cats.

Environmental dust cloth samples (floor, wall, feeding tray and toy) were collected daily from surfaces and analyzed by total E gene PCR. Viral RNA could be detected until the end of the study with the highest number of copies on the feeding dish samples. SARS-CoV-2 E gene RNA could furthermore be detected in air samples collected from the animal room on DPI 2, 4, 5, 6, 9 and 10, as well as in exhalation air samples collected from some, but not all, cats during DPI 2–11 (Supplemental Figure S2).

### 3.3. SARS-CoV-2 Mutation in Cats and Environment

From the three inoculated cats, who completed the full study until DPI 14, SARS-CoV-2 frequencies of alternative variants over time were analyzed using sequences data of swabs, tissue and environmental samples. SARS-CoV-2 showed mutations in all three cats and their environment, with a total of 1766 variants being called within the 78 samples (Supplemental Figure S3). The variants were equally distributed over the genome and only 230 of the variants had an alternative frequency above 25%. A mutational peak seemed to be present around bp 5750, but on closer inspection, there seemed to be a small variability hotspot with three mutations that occurred in higher frequencies (bp 5694, 5784 and 5849). When looking at the patterns over time on these positions, no dominant or mutations on their way to fixation were observed.

### 3.4. Humoral and Cellular Immune Responses

**Figure 3:**
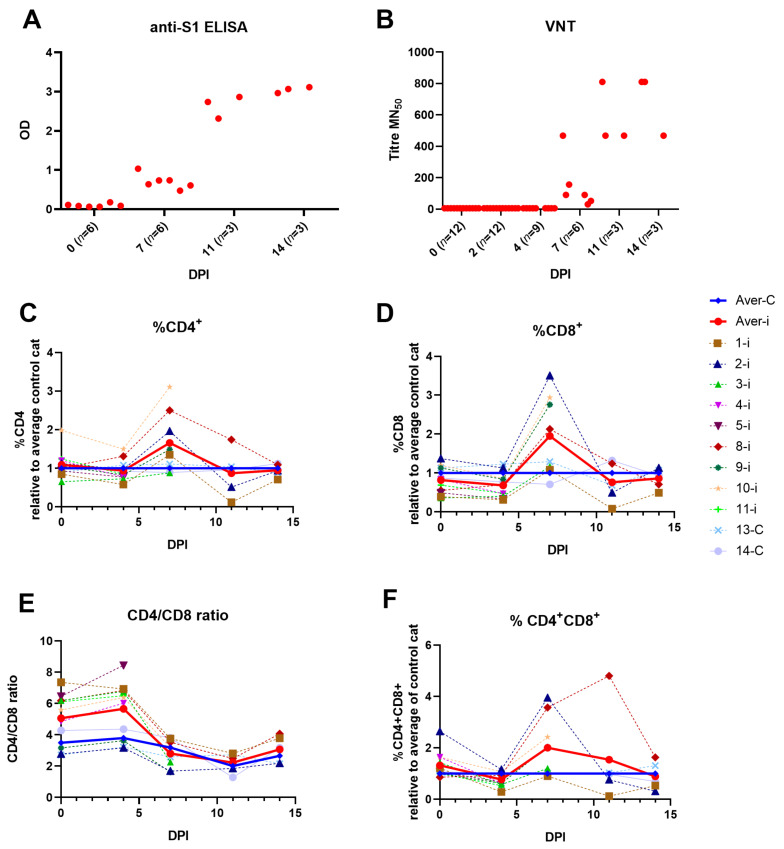
Humoral and cellular immune response after SARS-CoV-2 infection. (**A**) Anti-S1 antibody response measured by ELISA at OD_450._ (**B**) Neutralizing antibody titers expressed as 50% neutralization titer (MN_50_). (**C**–**F**) Whole blood was analyzed by flow cytometry for % of CD4+, CD8+, CD4+CD8+ cell and CD4/CD8 ratio on DPI 0, 4, 7, 11 and 14. The percentage (%) of the different subsets of the infected animals are shown as relative expression to average of control animals (13 and 14); (**C**–**F**) Infected animals euthanized on DPI 2 were not included. Average = Aver, infected = i, control = C. The number of infected animals decreased from DPI 7 due to euthanasia: DPI 0 (*n* = 9), DPI 4 (*n* = 9), DPI 7 (*n* = 6), DPI 11 and 14 (*n* = 3).

Sera and EDTA blood were collected on DPI 0, 2, 4, 7, 11 and 14. SARS-CoV-2 specific antibodies (anti-S1) and virus neutralizing antibodies (Figure 3A,B) were present in sera of all infected cats from DPI 7 with increasing levels until the end of the study.

The percentage of CD4+, CD8+ and CD4+CD8+ cell subsets and CD4/CD8 ratio in whole blood were measured after lysis of erythrocytes at DPI 0, 4, 7, 11 and 14. The percentages of the different subsets from the infected animals were compared to those of the control animals. Animals euthanized on DPI 2 were not included. Overall, there was individual variation for the different subsets. Only on DPI 7, there was an increase of the percentage of CD4+ and CD8+ cells in infected animals compared to control animals, which was not observed at later time points post infection (Figure 3C,D). On DPI 7, there was also a decrease of the CD4/CD8 ratio in infected animals compared to the ratio before infection, indicating an increase of the CD8+ subset compared to the CD4+ subset (Figure 3E). For the percentage of CD4+CD8+ cells in infected animals compared to control animals, there was a large variation between the animals with a tendency for a higher percentage of CD4+CD8+ cells on DPI 7 and 11 (Figure 3F).

On DPI 14, PBMCs from the remaining infected (*n* = 3) and control animals (*n* = 2) were used to measure specific SARS-CoV-2 IFN-gamma responses by ELISpot. No antigen specific IFN-gamma response could be detected on DPI 14 after in vitro stimulation with spike protein (SP), nucleoprotein (NP) or a peptide pool (Peptides) (Supplemental Figure S4). Non-stimulated PBMCs (medium control) of infected animals showed a higher basal IFN-gamma response compared to the control animals; however, this difference was not observed after ConA stimulation (positive control). On PBMCs obtained at DPI 7 and DPI 14, we also analyzed the percentage of CD4+, CD8+, CD4+CD8+ cells and CD4/CD8 ratio before and after stimulation with SP. In vitro restimulation did not result in differences in the percentages or ratio before and after stimulation in infected and control animals (Supplemental Table S4).

### 3.5. RNA Sequence Analysis in CD4+ and CD8+ Subsets at Different Time-Points after SARS-CoV-2 Infection

**Figure 4:**
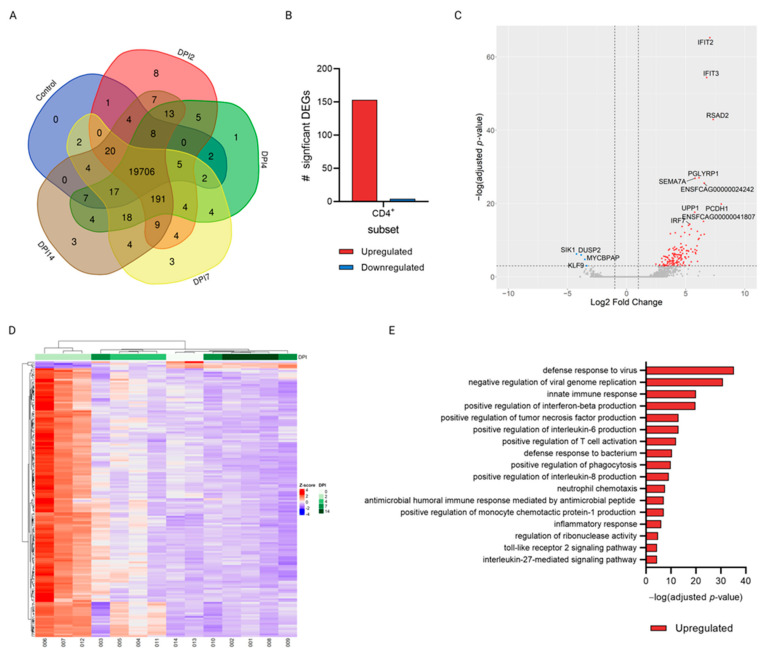
Differential gene expression analysis of the CD4+ subset after SARS-CoV-2 infection in cats. (**A**) Total number of unique or overlapping genes expressed at each timepoint, generated using the Venn tool developed by UGent [34]. (**B**) Total number (#) of significantly differentially up- (red) or down- (blue) regulated genes at 2-days post infection (DPI 2) compared to control animals as determined by DESeq2. (**C**) Volcano plot of all genes expressed at DPI 2 vs. control; red = upregulated; blue = downregulated, the unannotated genes shown as Ensembl ID of *Felis catus* genes (ENSFCAG) are specified in Supplemental Table S5. (**D**) Heatmap depicting the expression (represented as z-scores of normalized counts) of all significantly differentially expressed genes at each DPI compared to uninfected controls. (**E**) Significantly enriched biological processes in the dataset of upregulated genes at DPI 2 vs. control. Genes were considered significantly differentially expressed if the B-H-adjusted *p*-value was ≤0.05 and they had a L2FC of |≥2|; biological processes were considered significantly enriched if the B-H-adjusted *p*-value was ≤0.05.

**Figure 5:**
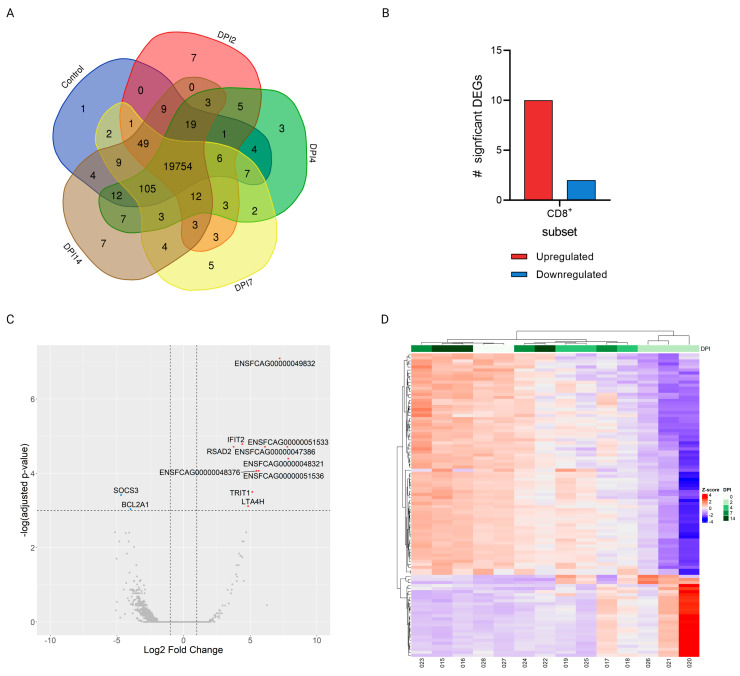
Differential gene expression analysis of the CD8+ subset after SARS-CoV-2 infection in cats. (**A**) Total number of unique or overlapping genes expressed at each timepoint, generated using the Venn tool developed by UGent [34]. (**B**) Total number (#) of significantly differentially up- (red) or down- (blue) regulated genes at DPI 2 compared to control animals as determined by DESeq2. (**C**) Volcano plot of all genes expressed at DPI 2 vs. control; red = upregulated; blue = downregulated, the unannotated genes shown as Ensembl ID of *Felis catus* genes (ENSFCAG) are specified in Supplemental Table S5. (**D**) Heatmap depicting the expression (represented as z-scores of normalized counts) of the top 100 differentially expressed genes at each DPI compared to uninfected controls. Genes were considered significantly differentially expressed if the B–H-adjusted *p*-value was ≤0.05 and they had a L2FC of |≥2|.

Gene expression of CD4+ and CD8+ subsets of infected cats at different time-points after SARS-CoV-2 infection (DPI 2, 4, 7, and 14) was compared with the expression in uninfected control animals. RNA sequencing on the Illumina NovaSeq6000 generated an average of 60 M paired-end 150 bp reads per sample. An average of 58M reads per sample remained after removal of adapter sequences and reads with a Q-score < 30 using TrimGalore [22]. In total, between 19,778–20,015 (average = 19952) and 19,875–20,000 (average = 19,954) genes were detected at the different timepoints within the CD4+ and CD8+ subsets, respectively. The majority of these were shared between the timepoints, with only a low number of genes unique to any timepoint (Figure 4A and Figure 5A). Using DESeq2 [27] for differential expression analysis of genes at different DPIs compared to uninfected controls, a total of 157 and 12 significantly differentially expressed genes (DEGs) were found in the CD4+ and CD8+ subsets, respectively (Figure 4B and Figure 5B). All of the significant DEGs detected were found in the comparison of DPI 2 vs. control in both subsets, whereas no genes were significantly differentially expressed in any of the other, later, timepoint comparisons. The distribution of the significant genes according to their L2FC and the negative log-transformed Benjamini–Hochberg (B-H)-adjusted *p*-values is visualized in Figure 4C and Figure 5C.

Several genes related to antiviral processes were found to be upregulated. In both subsets IFIT2 and RSAD2 related to interferon induction of antiviral activity were significantly upregulated, whereas related genes IFIT3 and IRF7 were significantly upregulated only in the CD4+ subset. Additionally, SEMA7A, involved in modulation of T-cell mediated responses in humans [32], and PGLYRP1, involved in antiviral activity [33], were significantly upregulated in the CD4+ subset. Furthermore, in the CD8+ subset, LTA4H, related to proinflammatory leukotriene B4 activation, was significantly upregulated, whereas SOCS3, which is involved in negative regulation of cytokines, was downregulated in this subset. Several unannotated genes were also found to be significantly differentially expressed (shown as Ensembl ID of *Felis catus* genes (ENSFCAG) in Figure 4C and Figure 5C). These were all novel, protein coding genes and are listed in Supplemental Table S5.

Heatmaps of all significant DEGs in the CD4+ subset show that samples of each timepoint clustered well together, apart from those of DPI 7 (Figure 4D). In the CD8+ subset, evaluating the top 100 DEGs, only the samples belonging to the DPI 2 and control groups clustered well within their groups (Figure 5D). Additionally, in both subsets, the expression patterns of the depicted genes at DPI 2 were clearly distinct from those seen in the controls.While, as time progressed, the gene expression patterns more closely resembled what was observed in uninfected cats (Figure 4D and Figure 5D). Functional annotation analysis of the significantly DEGs detected at DPI 2 vs. control in the CD4+ subset revealed that the biological processes (BP) enriched in our dataset of upregulated genes mostly related to innate immune responses and host defense against viral infection (Figure 4E). Similarly, although not significant, the only biological process enriched in the dataset of the CD8+ subset was the defense response to virus (data not shown). In both subsets, the few genes that were significantly downregulated at DPI 2 vs. control were not associated with any significantly enriched BPs.

## 4. Discussion

Cats are susceptible to SARS-CoV-2 infection and show in general no to only mild clinical signs, as reported in several field and experimental studies reviewed and summarized by [8,35,36]. However, there is limited information available about the immune response shortly after infection, which is necessary to strengthen our knowledge about the pathogenesis of SARS-CoV-2 in cats. This study describes the immune responses in cats after experimental SARS-CoV-2 inoculation and provides pathological characterization and data on infection kinetics following infection with Dutch SARS-CoV-2 isolate. After intranasal inoculation the young-adult cats developed no clinical signs and displayed only a mild transient increase in body temperature at DPI 2. A viral antigen was only expressed in the submucosal glands of the lung and trachea and in epithelial cells of nasal conchae and larynx associated with mild to moderate rhinitis and inflammation of submucosal glands in trachea and lung from DPI 2 to DPI 7. There was no viral antigen expression nor SARS-CoV-2 related histological changes in other investigated organs, such as intestine. Our clinical and histopathologic results were in line with the majority of the feline SARS-CoV-2 studies [6,9,37]. However, more severe lung pathogenicity characterized by interstitial pneumonia with diffuse alveolar damage has been observed as well [38,39]. One of these studies [39] described also viral expression in the intestinal tract with associated histological changes and diarrhea with weight loss in cats. These differences in pathogenicity and virus expression for lung and intestine could be related to the higher inoculation dose and/ or different SARS-CoV-2 strains, amongst other differences in experimental design such as inoculation route and age of animals. For instance, intratracheal inoculation of SARS-CoV-2 induced clinical respiratory signs in cats and severe lung pathogenicity similar to hospitalized humans [40,41]. Next to the inoculation route, the age of the cat is of importance. Shi et al. [38] showed that juvenile cats (less than 3 months of age) are more susceptible to SARS-CoV-2, with more severe lung pathogenicity on DPI 3, compared to young-adult cats as we used in this study. Additionally, old cats, diseased cats and immunosuppressed cats (for instance infected with feline immunodeficiency virus (FIV) or feline leukemia virus (FeLV)), could be more susceptible to SARS-CoV-2 as reported in some case reports [42,43,44].

Viral shedding from nose, mouth and or intestine is important for viral transmission. For detection of viral RNA, we used the E-gene PCR. Positive samples were further analyzed by sgPCR, which specifically quantifies newly transcribed viral subgenomic RNA of the E gene that is not packaged into virions. Therefore, sgRNA transcription can be interpreted as indicative of active virus replication [15,45]. From DPI 2 until DPI 7 all throat and nasal swabs from infected animals contained sgRNA copies. In line with this, infectious virus was isolated from respiratory tissue (nasal conchae, trachea, and lungs) between DPI 2 and 7. The observation that feeding dishes had high levels of viral RNA (E-gene PCR) as well as that viral RNA could be detected in exhalation air samples further emphasizes the oral and upper respiratory shedding route. In contrast, there was only limited sgRNA in colon/fecal samples, indicating that viral replication in the intestinal tract and thus shedding via feces is less abundant than nasal shedding. Our virology results are in line with other feline studies [9,37,38,39], where there is clear viral shedding in the time frame from shortly after inoculation until DPI 7, while at later time points the presence of viral RNA is only minimal.

Next to viral shedding, we also investigated the potential for host adapted mutations of the inoculated SARS-CoV-2 virus within cats and environment. Animals that can get infected with SARS-CoV-2 and subsequently infect other animals, can potentially be a virus reservoir in which virus circulates and could acquire mutations over time [7,46]. In our study we observed only minimal mutations over time. None of these mutations could be identified as biologically relevant, in the sense that it affected virus infectivity or replication. This is in contrast to a recent paper [16], where the authors observed species and context specific virus adaptations in a SARS-CoV-2 domestic cat model. This difference could be explained by similar reasons as mentioned above, such as different age or genetic background of cats, different inoculation dose and/or strains and different duration of the observation period.

SARS-CoV-2 infected cats developed an effective immune response, which resulted in rapid viral clearance, limited histopathological changes only in the respiratory tract and absence of clinical signs. Inoculated cats seroconverted starting on DPI 7 and developed specific virus neutralizing antibodies, which indicates an effective humoral immune response. The increase of CD4+ and CD8+ subsets and CD8/CD4 ratio in blood compared to uninfected animals, represented an activation of the cellular immune response with an higher increase of CD8+ cells (most likely cytotoxic T cells) compared to CD4^+^ T-helper cells. To investigate the SARS-CoV-2 specific cellular immune response in more depth, CD4+ and CD8+ subsets within PBMCs of infected animals on DPI 2, 4, 7 and 14 were analyzed for gene expression and compared with uninfected cats. Only at DPI 2, both subsets (although much more pronounced in the CD4+ subset than in the CD8+ subset) showed discernible changes in gene expression. Especially, in the CD4+ subset genes related to antiviral immune responses, IFN stimulation, (e.g., IFIT2, IFIT3 and RSAD2) were significantly upregulated. On DPI 4 changes in gene expression were negligible, indicating that the SARS-CoV-2 infection was rapidly cleared in infected cats and that gene expression patterns return to ‘baseline’ as observed in the uninfected controls. This rapid viral clearance most likely also affected the induction of a specific IFN-gamma response, which was not detected on DPI 14. Further, restimulation of PBMCs 7 and 14 days after infection with SARS-CoV-2 spike protein did not result in an increase in CD4+ and CD8+ subsets within the PBMCs. Due to this we speculate that the overall early immune response was effective enough and that further development of specific IFN-gamma producing T-cells was not required. 

The results reported in this study should be considered in light of several limitations: As for most studies involving cats, the low number of experimental animals is a major limitation. Not only for ethical reasons, but also in terms of husbandry and animal handling, studies involving a large number of cats are challenging, especially at biosafety level 3, and therefore the number of animals needed to be minimized. Moreover, this study was conducted early in the SARS-CoV-2 pandemic, and at that time, it was unclear whether there would be sex-associated differences with regards to susceptibility, clinical disease, immunology and pathology. We therefore chose both males and females for this study, and selected two females as uninfected control animals, because we prioritized sex-separated co-housing of cats over an equal distribution of both sexes in the experimental design. A third limitation of this study was the breed of cats which could influence susceptibility to virus infections, as reported before for feline infectious peritonitis virus, also a feline coronavirus, [47,48] and as well as the age of cats as described above. For this study, we selected SPF domestic shorthair cats; however in the field there are many other breeds and genotypes that were not considered in this study. Moreover, cats in the field may have or recently had an infection with another pathogen leading to an activated immune system or presence of cross-reacting antibodies that can influence the outcome of a SARS-CoV-2 infection. Finally, the collection of samples under general anesthesia during the first week post-inoculation could negatively impact an animal’s immune system [49,50]. Despite these limitations, we consider our results valuable, as they provide insights in the rapid clearance of a SARS-CoV-2 infection of healthy young-adult cats.

In conclusion, young-adult cats infected with SARS-CoV-2 developed a strong antiviral immune response shortly after infection, as shown by a large number of antiviral DEGs on DPI 2 in the CD4+ subset in blood, increased percentages of CD4+ and CD8+ cells in the blood on DPI 7 and the presence of virus-neutralizing antibodies on DPI 7. This ensured a rapid viral clearance within the first week after infection, without development of clinical signs. Furthermore, SARS-CoV-2-associated histopathological lesions in the lungs, trachea and nose were mild and only transient, and no relevant virus mutations indicative of host adaptation could be detected. 

## Data Availability

The data presented in this study are openly available in the Sequence Read Archive (SRA) of the National Center for Biotechnology Information (NCBI), at https://www.ncbi.nlm.nih.gov/bioproject/942620, under BioProject ID PRJNA942620.

## Supplementary Materials

The following supporting information can be downloaded at: https://www.mdpi.com/article/10.3390/v15051052/s1: Figure S1: Study overview; Figure S2: Viral RNA load (E-gene PCR) in tissues and environment after SARS-CoV-2 infection in cats; Figure S3: Frequency of alternative variants compared to the reference strain SARS-CoV-2; Figure S4: SARS-CoV-2 IFN-gamma responses on DPI 14; Figure S5: Health monitoring report of cats; Table S1: Group overview animal experiment; Table S2: Overview clinical parameters and virology; Table S3: histological changes and viral antigen expression; Table S4: PBMC restimulation 7 and 14 days after SARS-CoV-2 infection; Table S5: Overview unannotated genes CD4+ and CD8+ subset.
