# Supplementary material for: Immune Responses and Pathogenesis following Experimental SARS-CoV-2 Infection in Domestic Cats
Source: Viruses. 2023 Apr 25;15(5):1052. doi: 10.3390/v15051052 (PMC10222871; doi:10.3390/v15051052)
Supplement: Supplementary file 1 [file viruses-15-01052-s001.zip › supplementary materials/viruses-2340528-supplementary-2.pdf]

Supplemental Materials

Table S1. Group overview of the animal experiment.

Experimental group	Number of animals at start	Sex (animal IDs)	Inoculation		Necropsy schedule study day and animal IDs
Infected cats	12	7 male (ID 1-7); 5 female (ID 8-12)	0.5mL SARS-CoV-2, $10^{4.5}$ $TCID_{50}$		DPI 2: 6, 7 and 12<br>DPI 4: 4, 5 and 11<br>DPI 7: 3, 9 and 10<br>DPI 14: 1, 2 and 8
Control cats	2	2 female (ID 13 and 14)	0.5mL PBS		DPI 14: 13 and 14

*DPI (days post-infection); ID (identification number).

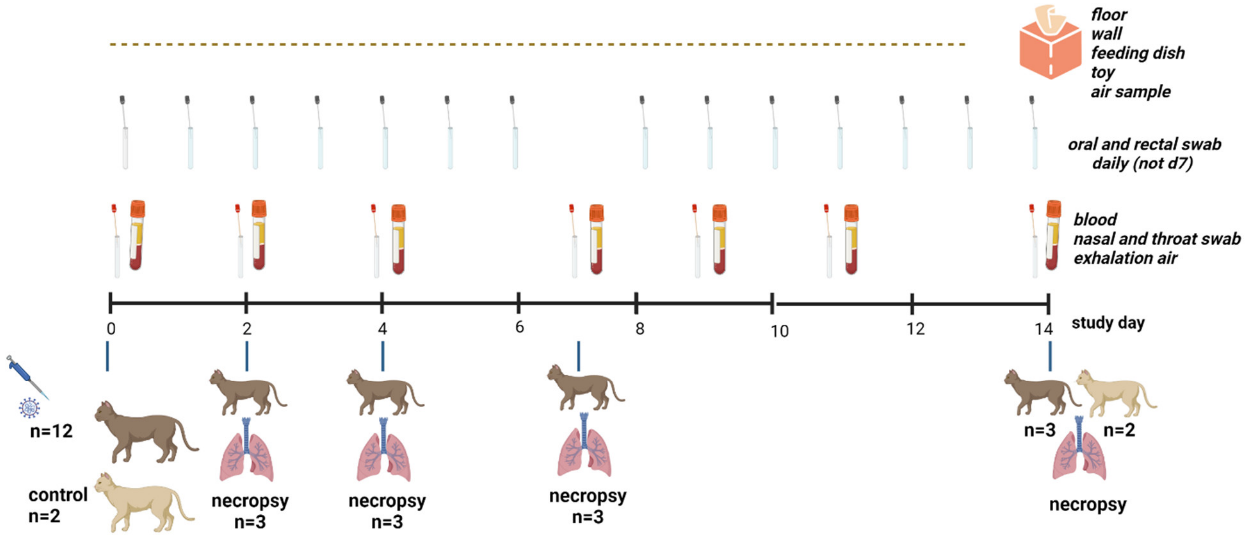

Figure S1. Study design. Study overview from inoculation (study day 0) until end of study (study day 14).

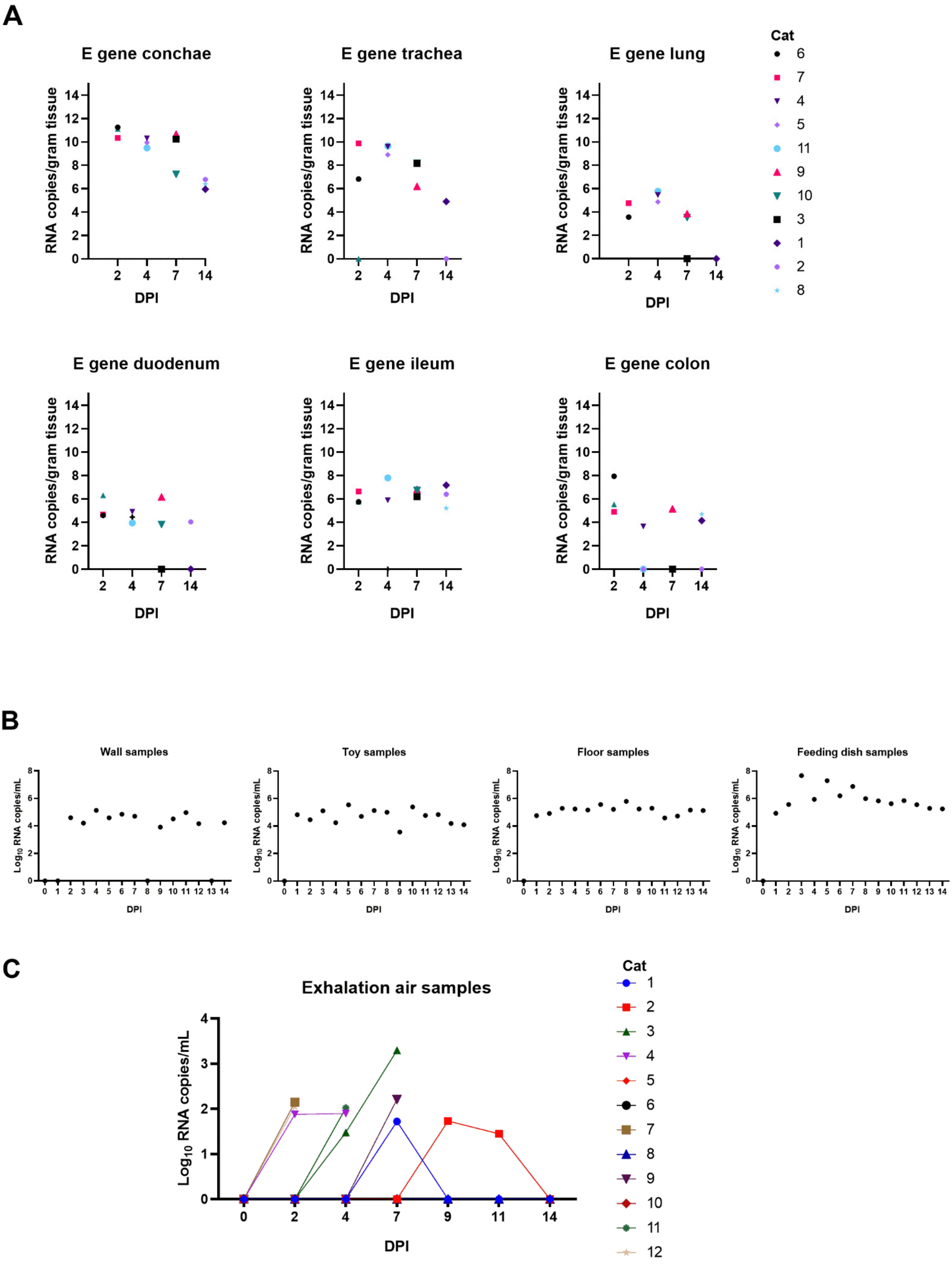
A
E gene conchae
E gene trachea
E gene lung
E gene duodenum
E gene ileum
E gene colon
RNA copies/gram tissue
DPI
Cat
6
7
4
5
11
9
10
3
1
2
8
B
Wall samples
Toy samples
Floor samples
Feeding dish samples
Log10 RNA copies/mL
DPI
C
Exhalation air samples
Log10 RNA copies/mL
DPI
Cat
1
2
3
4
5
6
7
8
9
10
11
12

Figures S2. Viral RNA load (E-gene PCR) in tissues and the environment after SARS-CoV-2 infection in cats. **(A)** Viral RNA (RNA copies/gram tissue) measured in tissues sampled during necropsy on days post-infection (DPI). All samples from the salivary gland were negative and not displayed. **(B)** Viral RNA (RNA copies/gram tissue) measured on the wall, floor, toy samples and feeding dishes. **(C)** Exhalation air samples of cats taken under anaesthesia on DPI 0, 2, 4, 7, 9, 11 and 14. Cat 1, 2 and 8 (DPI 0–14); cat 3, 9 and 10 (DPI 0–7); cat 4, 5 and 11 (DPI 0–4) and cat 6, 7 and 12 (DPI 0–2). Control animals 13 and 14 were negative and not included in the graph.

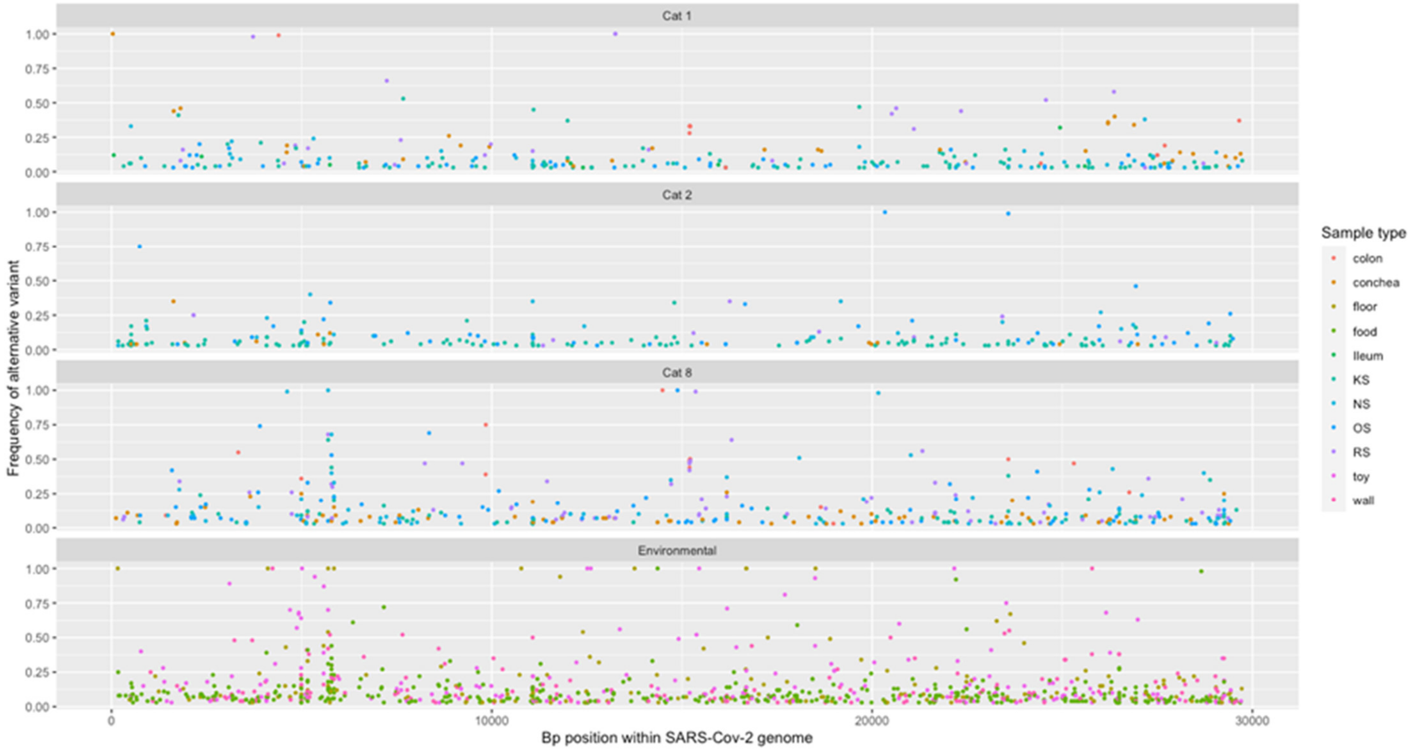

Figure S3. Frequency of alternative variants compared to the reference strain SARS-CoV-2. One facet per cat and one with the variants found in the environmental samples. Dots are colored by sample type. Abbreviations: KS = throat swab, NS = nasal swab, OS = oral swab and RS = rectal swab.

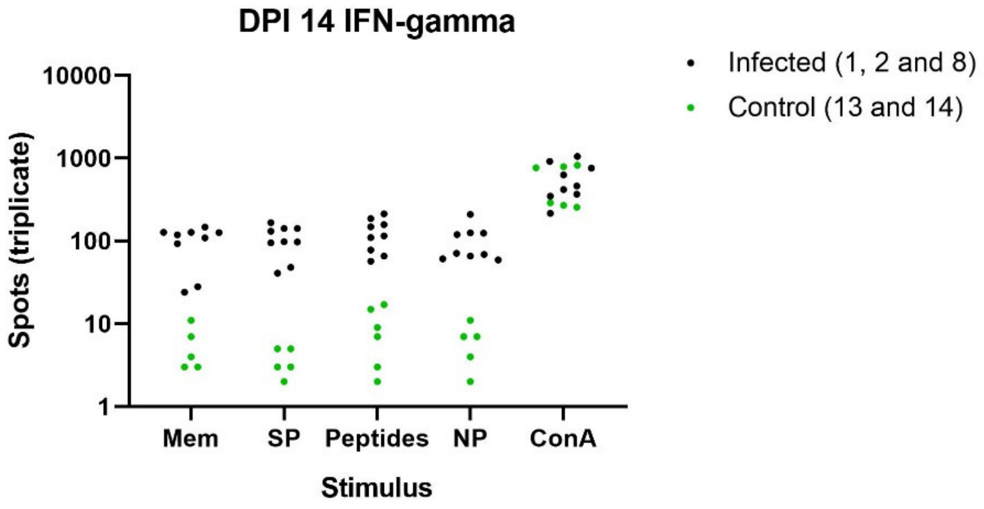

Figure S4. SARS-CoV-2 IFN-gamma responses on DPI 14. Interferon gamma response of PBMCs after stimulation with a spike protein (SP), nucleoprotein (NP), a peptide pool (peptides), Concanavalin A (ConA) and medium (Mem) as the control, measured by the ELISpot analysis.

Health Monitoring Report

Based on FELASA Recommendations

MARSHALL BioResources

Name and address of the breeder: [redacted]

Date of issue: December 2019 **Unit N°:** Barriers and Conventional Buildings **Examination date:** Quarter 4, 2019

Species: Feline **Breed:** Domestic Shorthair Cat **Populated†:** Colony acquired June 2018

	------BARRIER BUILDINGS------				-------CONVENTIONAL BUIDLINGS-------					
	1st BARRIER		2nd BARRIER		[redacted]		[redacted]			
	CUM	CURR	CUM	CURR	CUM	CURR	CUM	CURR	LAB	METHOD
**VIRAL INFECTIONS**										
**Compulsory Agents:**										
Feline Calicivirus	0/50	0/10	0/50	0/10	Vaccinated	NA	Vaccinated	NA	[redacted]	PCR (Oropharyngeal swab)
Feline Coronavirus	0/50	0/10	0/50	0/10	0/40	NE	0/30	0/10		PCR (Whole blood)
Feline Coronavirus	0/40	0/10	0/40	0/10	0/30	NE	0/30	0/10		PCR (Fecal)
Feline Herpesvirus	0/50	0/10	0/50	0/10	Vaccinated	NA	Vaccinated	NA		PCR (Conjunctival swab)
FeLV/FIV	0/50	0/10	0/50	0/10	0/40	NE	0/30	0/10		ELISA (Whole blood)
Feline Panleukopenia	0/50	0/10	0/50	0/10	Vaccinated	NA	Vaccinated	NA		PCR (Feces)
Feline Panleukopenia	0/50	0/10	0/50	0/10	Vaccinated	NA	Vaccinated	NA		HI (Serum)
**BACTERIAL INFECTIONS**										
**Compulsory Agents:**										
Bartonella spp.	0/50	0/10	0/50	0/10	0/40	NE	0/30	0/10	[redacted]	PCR (Whole Blood)
Bordetella bronchiseptica	21/50	4/10	22/50	1/10	15/40	NE	18/30	4/10		Culture (Oropharyngeal swab)
Campylobacter spp.	0/50	0/10	0/50	0/10	0/40	NE	0/30	0/10		Culture (Rectal swab)
Chlamydophilia	0/50	0/10	0/50	0/10	0/40	NE	0/30	0/10		PCR (Conjunctival swab)
Mycoplasma haemofelis	0/50	0/10	0/50	0/10	0/40	NE	0/30	0/10		PCR (Whole Blood)
Mycoplasma Culture	0/50	0/10	0/50	0/10	0/40	NE	0/30	0/10		Culture (Oropharyngeal swab)
Pasteurellaceae	34/50	9/10	3/50	0/10	36/40	NE	28/30	8/10		Culture (Oropharyngeal swab)
Salmonella spp.	0/50	0/10	0/50	0/10	0/40	NE	0/30	0/10		Culture (Rectal swab)
Streptococci β-hemolytic Group G	19/50	2/10	16/50	2/10	11/40	NE	8/30	5/10		Culture (Oropharyngeal swab)
Yersinia enterocolitica	0/50	0/10	0/50	0/10	0/40	NE	0/30	0/10		Culture (Rectal swab)
**FUNGAL INFECTIONS**										
**Compulsory Agents:**										
Microsporum spp.	0/50	0/10	0/50	0/10	0/40	NE	0/30	0/10	[redacted]	Fungal Culture (Hair pluck)
Trichophyton spp.	0/50	0/10	0/50	0/10	0/40	NE	0/30	0/10		Fungal Culture (Hair pluck)
**PARASITOLOGICAL**										
**Compulsory Agents:**										
Helminths	0/50	0/10	0/50	0/10	0/40	NE	0/30	0/10	[redacted]	Sodium Nitrate (Fecal)
Isospora spp.	5/50	0/10	5/50	0/10	2/40	NE	0/30	0/10		Sodium Nitrate (Fecal)
Sarcocystis spp.	0/50	0/10	0/50	0/10	0/40	NE	0/30	0/10		Zinc Sulfate (Fecal)
Toxoplasma gondii	0/50	0/10	0/50	0/10	0/40	NE	0/30	0/10		IgG IFA (Serum)
**Agents on Request:**										
Giardia sp.	0/10	NE	0/10	NE	0/10	NE	NE	NE	[redacted]	Zinc Sulfate (Fecal)

NA=not applicable CUM=cumulative
NE=not examined CURR=current

†Colony was established by Liberty Research in 1975, and acquired by Marshall in June 2018. Conventional animals originated from barriers.

The vaccination program is administered in accordance with current veterinary practice procedures and is documented accordingly. A description of the current immunization regimen is available upon request.

Abbreviations for laboratories:

[redacted]

[redacted] 12/20/2019
Date

Figure S5. Health monitoring report of the cats.
